# Atypical Femur Fractures Without Bisphosphonate Exposure (AFFwB): A Retrospective Report of 21 Cases

**DOI:** 10.3390/jcm15010025

**Published:** 2025-12-19

**Authors:** Lorenzo Lucchetta, Carmelinda Ruggiero, Samuele Berardi, Alice Franceschi, Michele Bisaccia, Giuseppe Rinonapoli

**Affiliations:** 1 Orthopedics and Traumatology Department, Medical School, University of Perugia, S. Maria della Misericordia Hospital, 06123 Perugia, Italy; 2 Orthogeriatric and Geriatric Units, Gerontology and Geriatrics Section, Department Medicine and Surgery, Geriatric Institute, Medical School, University of Perugia, S. Maria della Misericordia Hospital, 06123 Perugia, Italy; 3 Orthopedics and Traumatology Department, Università Cattolica del Sacro Cuore, 00168 Rome, Italy

**Keywords:** atypical femoral fracture, bisphosphonates, osteoporosis, vitamin D, bone turnover, principal component analysis

## Abstract

**Background/Objectives:** Atypical femoral fractures (AFFs) are rare and classically linked to long-term bisphosphonate therapy, but many occur without such exposure. We aimed to characterize atypical femoral fractures without bisphosphonate exposure (AFFwB) in older adults and to explore biochemical patterns using principal component analysis (PCA). **Methods:** We conducted a retrospective study of patients ≥65 years admitted with femoral fractures (January 2019–September 2024). AFFs were identified from ICD-9 codes and confirmed according to 2014 ASBMR criteria by two blinded reviewers. Demographic, clinical, densitometric, and metabolic data were collected. Correlations between biochemical variables were assessed, and PCA was applied to PTH, vitamin D, BMD, ALP, calcium, and creatinine. **Results:** Among 932 femoral fractures, 36 met AFF criteria, including 21 AFFwB cases. AFFwB patients were mostly women, and fractures were predominantly diaphyseal. Vitamin D insufficiency/deficiency and osteoporosis were observed in 77% and 66.7% of cases, respectively. Strong correlations were found between PTH and vitamin D and between PTH and ALP. PCA identified four components explaining 88.2% of variance, corresponding to endocrine–mineral regulation, bone turnover, renal function, and BMD as an independent domain. Exploratory comparison with bisphosphonate-exposed AFF suggested higher vitamin D levels and lower ALP in treated patients. **Conclusions:** In this cohort, AFFs occurred both in patients without bisphosphonate exposure and in those receiving antiresorptive therapy, indicating that AFFs may arise through different pathways. These findings suggest that both insufficient bone quality and excessive remodeling suppression can ultimately lead to atypical fractures. Further studies are needed to clarify these findings.

## 1. Introduction

Atypical femoral fractures (AFFs) are rare, low-energy fractures that occur in the subtrochanteric or diaphyseal region of the femur. They are described by the American Society for Bone and Mineral Research (ASBMR) criteria to distinguish AFFs from typical osteoporotic femur fractures. Key features are the minimal or no trauma, transverse or short oblique orientation, involvement of the lateral cortex, minimal comminution, and localized cortical thickening at the fracture site [1,2].

AFFs have been most reported in patients undergoing long-term treatment with bisphosphonates [3,4]. Although these drugs are effective in reducing osteoporotic fractures, prolonged use has been linked to impaired bone remodeling and microdamage accumulation, with an estimated incidence of 112 AFF per 100,000 patient-years after > 8 years of therapy [5,6]. Nonetheless, AFFs have also been observed in individuals not exposed to bisphosphonates [7,8], suggesting that other factors, including anatomical variations, genetic predisposition, or concurrent pharmacological treatments, may also play a role [5,9].

Therefore, the purpose of this study was (1) to analyze a series of AFFs occurring in patients without bisphosphonate exposure (AFFwB), comparing them with the AFFs with Bisphosphonate Exposure (AFFB); and (2) to investigate the potential role of other pharmacological agents, such as proton pump inhibitors and glucocorticoids, as well as anatomical and metabolic factors, and their influence on AFFs.

## 2. Materials and Methods

An observational retrospective study was conducted on all patients aged 65 years or older, admitted at the Department of Orthopedics and Traumatology, Santa Maria della Misericordia Hospital, Perugia, diagnosed with femoral fractures between January 2019 and September 2024.

The Orthopedics and Traumatology Department of Perugia have an average of 600 hip fractures per year according to AGENAS Italy (National Agency for Regional Health Services) [10], and their care management is done through an orthogeriatric team specialized in the treatment and care of geriatric patients over 65 with fragility fractures.

Two orthopedics surgeons’ residents (L.L and A.F.) examined the imaging electronical database of the Radiology of the Hospital and selected the patients with potential AFFs following the ICD-9-CM 820.22 (Closed fracture of subtrochanteric section of neck of femur), 821.01 (Closed fracture of shaft of femur) and 821.22 (Closed fracture of epiphysis, lower separation of femur); all the other ICD-9-CM Codes for proximal femoral fractures were excluded.

Then to confirm and identify AFFs, radiographic images of all included patients were reviewed using the 2014 American Society for Bone and Mineral Research (ASBMR) Task Force and labeled them with a numeric anonymous code for a blind evaluation by the senior orthopedic surgeon (G.R.) and the senior geriatrician (C.R.) each blinded one to another.

For each AFF included, medical records were screened by S.B. and A.F. to collect the data of demographics, trauma mechanism, presence of prodromal symptoms, comorbidities, history of previous fractures, clinical therapy, bone mineral density (BMD, T-score) and laboratory exams.

The patients who were not undergoing bisphosphonate therapy in the last 30 days, were included in the Atypical Femur Fractures without Bisphosphonate Exposure (AFFwB) group. The patients with AFFs who were under bisphosphonates therapy were included in the Atypical Femur Fractures with Bisphosphonate Exposure (AFFB) group.

The laboratory markers of bone metabolism collected and their reference values were: Parathyroid hormone (PTH): 15–65 pg/mL; Vitamin D: Deficiency < 10 ng/mL, Insufficiency 10–30 ng/mL, Sufficiency 30–100 ng/mL, Toxicity > 100 ng/mL; Alkaline phosphatase (ALP): 45–115 U/L (men), 55–142 U/L (women); Calcium: 8.9–10.1 mg/dL; Creatinine: 0.55–1.12 mg/dL

Missing clinical data were retrieved, when possible, through telephone interviews. Patients with incomplete medical records data were excluded.

Informed consent was obtained from all participants, and their rights were protected in accordance with ethical guidelines, CARE guidelines [11] were followed during the design of the study.

### Statistical Analysis

The evaluation of both the AFFwB and AFFB groups and a comparative assessment between them was planned. If a full comparative analysis was not feasible, each group was to be analyzed independently, and descriptive statistics were used to compare the two groups.

Continuous variables were summarized as mean, median, standard deviation, minimum, and maximum values, while categorical variables were reported as absolute and relative frequencies. The distribution of continuous variables was assessed using the Shapiro–Wilk test. Based on normality results, associations between biochemical and clinical variables were analyzed using Pearson’s correlation coefficient for normally distributed data and Spearman’s rho for non-normally distributed variables.

Missing data were handled using mean imputation for each variable prior to multivariate analysis. Principal Component Analysis (PCA) was performed to explore underlying patterns of covariation and to identify the variables contributing most to overall variance. Only variables of clinical relevance and available in most cases (PTH, Vitamin D, BMD, ALP, Calcium, and Creatinine) were included in the PCA model.

Sampling adequacy was assessed through the Kaiser–Meyer–Olkin (KMO) measure, while the suitability of the correlation matrix for factor analysis was evaluated using Bartlett’s test of sphericity. Components were extracted based on eigenvalues > 1 and the proportion of explained variance. A Promax oblique rotation was applied to allow for correlation among components and to optimize interpretability of factor loadings. A two-tailed significance threshold of *p* < 0.05 was adopted for all analyses. All statistical procedures were performed using R software 4.5.2 [12].

## 3. Results

**Figure 1:**
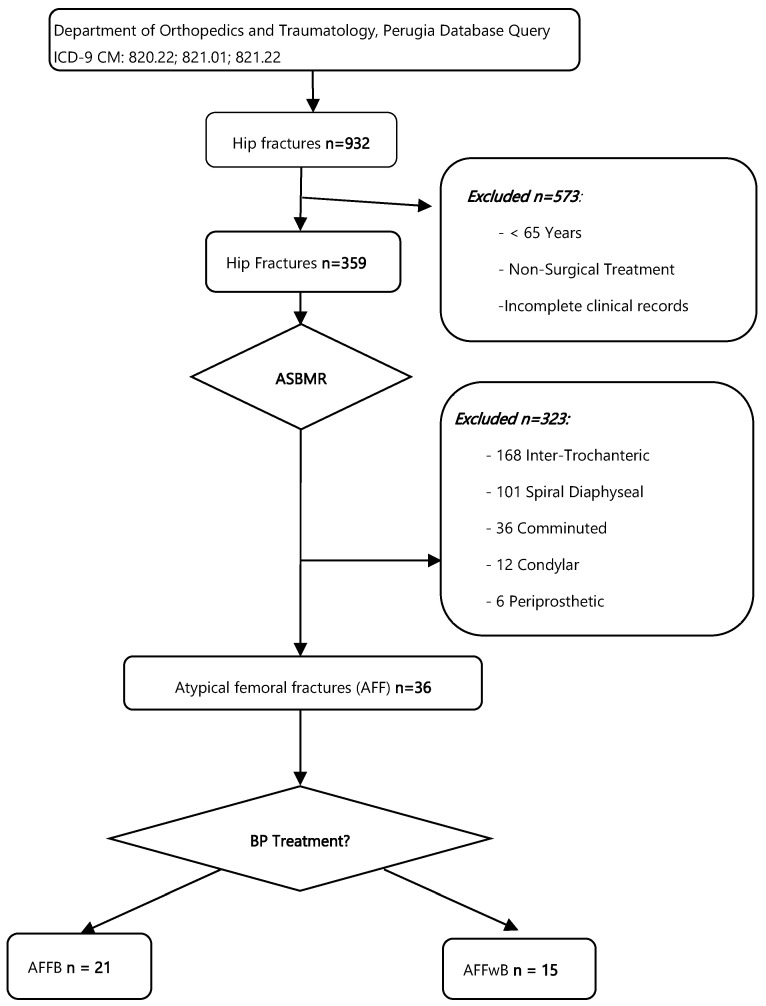
Patients Selection Flowchart. BP: Bisphosphonate.

A total of 932 subtrocantheric and diaphyseal femoral fractures were identified through the database ICD9 query (820.22, 821.01 and 821.22). Patients’ flowchart inclusion is shown in Figure 1.

By inspecting each patient surgical dossier 573 patients who were <65 years old, and non-surgical related admissions were excluded. 359 cases of hip and diaphyseal fractures remained.

The application of the ASBMR criteria on the radiographs, reviewed by the two blinded seniors’ examiners G.R and C.R, led to the exclusion of 323 patients due to the presence of intertrochanteric (n = 168), spiral diaphyseal (n = 101), comminuted (n = 36), mono and bicondylar (n = 12), or periprosthetic fractures (n = 6). Finally, 36 cases (3.86%) consistent with atypical femur fractures (AFF) were included.

By checking patients’ clinical dossiers, the patients who were receiving bisphosphonate treatment at the time of fracture or within the previous 30 days (n = 15) were included in the AFFB group, yielding 20 patients in the AFFwB group. One patient, who had never received bisphosphonate treatment but had started denosumab therapy within one year before the fracture (only a single administration) was included in the AFFwB group. A final cohort of 21 AFFwB patients was identified, representing 2.25% of all diagnosed femoral fractures. Of these, 17 patients were women and 4 men, with a mean age of 78.5 ± 8.92 years. Fractures were diaphyseal in 15 cases and Subtrocantheric in 6 cases. At the time of case review, all the fractures healed within 6 months from surgery and six patients (28.6%) had deceased.

All the demographic and laboratoristic data collected are shown in Table 1.

The AFFB group represented the 1.61% of all diagnosed hip fractures, but due to incomplete anamnestic, biochemical and densitometric data, was not suitable for a full statistical comparison against AFFwB. Only a descriptive summary of 6 patients was possible. These patients showed similar demographic characteristics to the main cohort, all women with a mean age of 79.8 ± 7.73 year.

### 3.1. Comorbidities

The most common comorbidity was hypertension, reported in 9 patients (42.9%).

Additional conditions identified within the cohort included diabetes mellitus (type 1 or 2), dyslipidemia, chronic kidney disease, ischemic heart disease, hypothyroidism, and chronic anemia. Other less frequent comorbidities comprised chronic ischemic encephalopathy, depression, dementia, gastritis, and psoriasis (see Table 1 for complete demographic and clinical details).

5 patients (23.8%) from the AFFwB group had no comorbidities at all.

At the time of fracture, 3 patients (14.3%) were receiving proton pump inhibitor therapy, although treatment duration was not available, and 1 patient (4.8%) was undergoing glucocorticoid therapy for COPD.

Within the AFFB subgroup, all patients presented both hypertension and osteoporosis. One patient had been under long-term glucocorticoid therapy (>20 years) for rheumatoid arthritis. Among this group, four patients were treated with alendronic acid (70 mg once weekly), one with clodronic acid (intramuscular, once weekly), and one with denosumab, who had prior bisphosphonate exposure before initiating denosumab therapy.

### 3.2. Prodromal Symptoms

Eight patients (38.1%) reported prodromal symptoms, primarily groin or thigh pain, in the month preceding the fracture. One patient reported experiencing functional impairment of the limb prior to the fracture, without any previous trauma. All fractures resulted from low-energy trauma, with the majority occurring after a fall from standing height. Three patients fell while walking, two indoors and one outdoors. One patient sustained the fracture while transitioning from sitting to standing, experiencing sudden and severe pain, while another fell from a seated position. For five patients, the exact mechanism of injury was not documented.

In the AFFB group, three patients reported no prodromal symptoms and only one experienced groin or thigh pain. For the remaining patients, it was not possible to determine the presence of prodromal symptoms.

### 3.3. Previous Fractures

More than half of the patients (57.1%) had no history of previous fractures. Three patients had previously undiagnosed vertebral fractures: one at D11, one at D12, and one at L1. Two patients had multiple vertebral fractures: one at D10 and D12, and the other at D7, D8, and D9. Only one patient had a contralateral hip fracture. In one case, data on previous fractures were not available.

In the AFFB group, half of the patients had history of previous fragility fractures like vertebral fractures and femoral fractures.

### 3.4. Laboratory Data

Vitamin D levels were insufficient in 17 patients (77%), with eight classifieds as deficient and nine as insufficient. Only two patients had sufficient Vitamin D levels. Parathyroid hormone (PTH) levels were elevated in 12 patients (55%), while nine had normal values.

All patients underwent a DEXA (Dual-Energy X-ray Absorptiometry) scan within six months of the fracture event. Osteoporosis was identified in 14 patients (66.7%), while three (14.3%) had osteopenia based on T-score values (T-score = −1.4, −1.5, −2.3).

Three patients (14.3%) were diagnosed with osteoporosis at the time of fracture. Among them, one was receiving Vitamin D supplementation only, and another was treated with Vitamin D, calcium, and denosumab. The third patient was not undergoing any osteoporosis treatment. None of them had received prior bisphosphonate therapy.

In the AFFB group, all patients were diagnosed with osteoporosis, and three of them were being treated with Vitamin D.

### 3.5. Statistical Analysis and Correlation Findings

The Shapiro–Wilk normality test revealed that BMD and Serum Calcium levels did not follow a normal distribution (*p* = 0.773 and *p* = 0.220) (Table 2). For this reason, correlation analyses were primarily performed using Spearman’s correlation coefficient. Spearman’s correlation analysis identified a strong negative correlation between PTH and Vitamin D (r of Pearson = −0.765, ρ of Spearman = −0.859; *p* < 0.001), a strong positive correlation between Vitamin D and Serum Calcium (r = 0.740, *p* = 0.001; ρ = 0.487, *p* = 0.056), and between PTH and ALP (r = 0.651, *p* = 0.005). A weak negative correlation between ALP and Serum Calcium (ρ = −0.638, *p* = 0.004) and a weak positive correlation between Creatinine and ALP (ρ = 0.510, *p* = 0.036) (Supplementary Table S1).

Principal Component Analysis (PCA) identified the key variables contributing most to total variance: PTH, Vitamin D, BMD, ALP, Calcium, and Creatinine. Missing data were handled using mean imputation (Table 3). The dataset demonstrated moderate sampling adequacy with a Kaiser-Meyer-Olkin (KMO) value of 0.445, while Bartlett’s test was statistically significant (χ^2^ = 29.8; gdl = 15; *p* = 0.013) (Table 4) PCA identified four principal components, collectively explaining 88.2% of the total variance (Table 5):

- PC1 (29.8%): Calcium metabolism and hormonal regulation, primarily influenced by Vitamin D (1.061), PTH (−0.441), and Serum Calcium (0.696);
- PC2 (21.6%): Bone turnover, correlated with ALP (1.050) and Serum Calcium (−0.415);
- PC3 (20.2%): Renal function and endocrine regulation, mainly influenced by Creatinine (0.946) and, to a lesser extent, PTH (0.552);
- PC4 (16.5%): Bone Mineral Density (BMD) as an independent factor, with a loading of 0.990.

### 3.6. AFFwB and AFFB Descriptive Comparison

Despite limited data availability, a descriptive comparison between AFFwB and AFFB was performed. AFFwB patients showed lower BMD values than those with AFFB (−3.17 vs. −2.37), indications a more severe osteoporosis in untreated individuals. Mean serum Vitamin D concentrations in patients with AFFB were significantly higher (33.9 vs. 15.2 ng/mL) and comparable PTH levels (66.5 vs. 71.6 pg/mL), though with greater variability. Moreover, alkaline phosphatase levels were lower in the AFFB group (71.0 vs. 89.2 U/L), consistent with the suppressed bone turnover induced by bisphosphonates. Mean creatinine levels were lower among AFFB cases (0.808 vs. 1.24 mg/dL), suggesting overall preserved renal function. Serum calcium values were comparable between groups (8.65 vs. 8.72 mg/dL), indicating a generally stable calcium–phosphate balance across both populations.

## 4. Discussion

Since their introduction into clinical practice, bisphosphonates have been widely used as a first-line treatment for osteoporosis due to their well-documented efficacy in reducing the risk of fragility fractures [8]. Although growing evidence has linked prolonged bisphosphonate use to an increased risk of atypical femoral fractures (AFFs), their overall risk-benefit balance continues to support their use as a first-line therapy in appropriate patients [5,8].

In the present cohort, 932 hip and diaphyseal femoral fractures were analyzed and 36 (3.86%) atypical femoral fractures (AFFs) were found, aligned with the overall low incidence described in the current literature [2,6].

In Italy, few studies have investigated this concept. Compared to another Italian study by Pedrazzoni et al. [13], our findings showed a modest discrepancy from the reported 7.1% incidence, which could be attributed to differences in study design particularly regarding patient age inclusion criteria of over than 40 Years old [13,14]. The present study instead, focused on a geriatric population over 65 Years, providing a sample more representative of the epidemiology of AFFs.

In the examined population, atypical femoral fractures not associated with bisphosphonate therapy (AFFwB) had an incidence of 2.25% among all femoral fractures. Notably, the literature reports that a percentage between 10% and 64.7% of AFFs occur in patients without bisphosphonate exposure [7,15,16].

Furthermore, several case reports have documented AFFs in patients never treated with bisphosphonates. Refs. [17,18] and this observation contributed to the removal of bisphosphonate exposure as a minor diagnostic criterion in the second ASBMR report [1,19].

Additional concerns have arisen from observations of a lower AFF incidence in patients treated with denosumab, another antiresorptive agent that also suppresses bone turnover [3,20]. In the present cohort, only one patient (4.5%) was receiving denosumab at the time of fracture.

Moreover, one of the critical challenges in AFF risk stratification is the lack of consensus regarding the definition of “long-term therapy”. Currently, there is no universally accepted duration beyond which bisphosphonate therapy significantly increases AFF risk or a clear indication for treatment suspension [21]. In this study, we considered treatment durations of ≥5 years as prolonged. This threshold is supported by literature suggesting an increased risk of AFF beyond this timeframe [6,8,9].

The etiology of AFFs is widely regarded as multifactorial. Therefore, to understand their pathogenesis, it is necessary to analyze not only the role of bisphosphonates but also the potential influence of other medications, such as glucocorticoids and proton pump inhibitors, whose associations with AFFs remain uncertain [22,23].

In the current analysis, one patient in the AFFwB group (4.5%) had been treated with both a PPI (esomeprazole) and glucocorticoids (prednisone) for five years. This result aligns with the findings from Juby et al., Kim et al., and Pedrazzoni et al., who reported glucocorticoid use in 12% to 20.7% of AFF cases [13,22].

A high prevalence of vitamin D deficiency was observed in the study population, with 77.3% of individuals showing insufficient serum levels [1,7,24,25]. The mean vitamin D concentration was 15.20 ± 11.80 ng/mL, comparable to the 16.8 ± 9.1 ng/mL reported by Gani et al. and 22.37 ± 5.6 ng/mL reported by Gani et al. [7,24,25]. Prodromal symptoms such as thigh or groin pain were reported by 36.4% of the patients. This prevalence is higher than the 27% reported by Juby et al. but lower than the 70% documented in the 2014 ASBMR report [1,26].

A history of previous fractures was reported by 40.9% of the individuals, suggesting a possible association with increased AFF risk. However, the literature shows substantial variability, with prevalence ranging from 18.6% to 35.3% [6,8,27].

With the increasing trend in prosthetic implantation, it becomes increasingly important to address the ongoing debate regarding the exclusion of periprosthetic fractures from AFF diagnostic criteria. According to Singh et al., the incidence of total hip and knee arthroplasty is projected to increase by 284% and 401%, respectively, by 2040 [28]. Recent studies, such as Huang et al. (2024), suggest that atypical periprosthetic femoral fractures share clinical and prognostic features closely consistent with those of AFF [29]. 

In this study, two cases of periprosthetic atypical femoral fractures were excluded according to ASBMR criteria. Nevertheless, multiple case reports [17,30] and reviews [5,31] have highlighted that these fractures often display radiographic and clinical patterns similar to AFF, supporting the hypothesis that their inclusion could expand case identification, enhance etiopathogenetic understanding, and guide the development of specific therapeutic algorithms [30]. These findings also support the potential etiological role of altered femoral geometry and mechanical stress concentrated on the lateral cortex [29].

Another notable finding in the present cohort was the absence of incomplete AFFs and contralateral femoral involvement. Incomplete AFFs are often underdiagnosed due to nonspecific symptoms (e.g., thigh or groin pain) and the lack of clear radiographic findings. Some publications have advocated for the use of DEXA scans to detect cortical thickening and early lateral cortical fracture lines [32]. Moreover, the management of incomplete AFF remains controversial. While conservative treatment may be an option, many authors advocate for prophylactic surgical intervention with anterograde intramedullary nailing in cases with a high risk of progression or persistent pain [33].

We hypothesize that, within our study, a diagnosis of fracture was not made when the fracture was incomplete. Nonetheless, an incomplete fracture could be present during the prodromal phase in some patients even if the diagnostic X-ray, was only conducted once the fracture had fully completed [2].

Regarding the literature on AFFS, most studies used traditional statistical methods, such as *t*-tests, chi-square, and regression models, to examine direct associations between variables and outcomes [6,8,34].

In contrast, this study applied Principal Component Analysis (PCA) to explore hidden patterns among biochemical and densitometric variables in the AFFwB group. Unlike most previous works [8], PCA revealed four independent physiological domains—endocrine regulation and mineral homeostasis (PC1, 29.8%), bone turnover activity (PC2, 21.6%), renal function influencing calcium-phosphate balance (PC3, 20.2%), and bone mineral density as an independent factor (PC4, 16.5%)—collectively explaining 88.2% of the total variance [35].

This analysis underscored the multifactorial nature of AFFwB, showing that alterations in Vitamin D and PTH impair bone remodeling and mineralization, that bone formation and mineralization may occur independently of bone density, and that renal dysfunction can increase cortical fragility. Overall, these results support the hypothesis that bone metabolism and bone mineral density represent distinct but complementary axes in the pathogenesis of bisphosphonate-unrelated AFF [34].

Given the considerable number of patients with AFFwB found in this study, it cannot be excluded that bisphosphonates may not be the primary cause of AFFs. Instead, severe osteoporosis itself could be the main responsible leading to fractures occurring in unusual locations. The fact that many reported cases involve patients who had been receiving bisphonate therapy for years may simply reflect that these individuals were already affected by advanced osteoporosis and were therefore undergoing such treatment. This distinction carries important clinical implications, emphasizing the need for targeted therapeutic strategies addressing both metabolic and structural aspects of bone health.

The preliminary descriptive findings of the comparison between AFFB and AFFwB may suggest that AFFwB patients show a pattern characterized by severe osteoporosis and increased bone turnover [36]. In contrast, AFFB cases represent a paradoxical phenomenon in which prolonged suppression of bone remodeling leads to microarchitectural alterations and reduced mechanical resistance, ultimately predisposing to atypical fractures despite near-normal BMD values.

The first limitation of this study lies in its retrospective observational design, which limited the completeness of clinical and laboratory data and may have introduced informational bias. Several key biochemical parameters were not routinely collected for all patients, resulting in missing data in a substantial subset of cases. In addition, the application of Principal Component Analysis (PCA) was limited by the relatively small sample size, which may have affected the robustness and generalizability of the identified component structure.

Another important limitation concerns the AFFB subgroup as, it was not possible to include these patients in the comparative statistical analysis with the AFFwB cohort, which was initially one of the primary objectives of this study. This could be partially explained, as most AFFB cases were retrieved from a period preceding the implementation of a standardized orthogeriatric management and data collection protocol. As a result, while diagnostic imaging data were available, essential clinical, biochemical, and densitometric information was incomplete or missing, preventing a meaningful comparative evaluation. This limitation underscores the need for a structured and systematic data collection framework, which we hope will serve as a foundation for future studies aiming to compare these two patient populations more effectively. Finally, given the rarity of AFF, the single-center nature of this study limits the generalizability of the findings. Expanding the research across multiple clinical centers would improve data completeness and enhance statistical power.

## 5. Conclusions

In this geriatric cohort, AFFs occurred both in patients treated with bisphosphonates and in those without prior exposure, indicating that AFFs may arise through different yet converging mechanisms. These findings suggest that both inadequate bone quality and excessive suppression of bone turnover can ultimately lead to atypical fractures. Larger, standardized multicenter studies are needed to better delineate these pathways and guide optimized preventive and therapeutic strategies.

## Figures and Tables

**Table 1:** Atypical Femur Fractures without Bisphosphonate Exposure (AFwB) Demographics—Pr.SY: Prodromal Symptoms; ALP: Alkaline phosphatase; Ca: Calcium; Cr: Creatinine.

N	Age/ Sex	Fracture	Side	Pr.SY	Comorbidities	Previous Fractures	BMD	Vit D	PTH	ALP	Ca	Cr
1	81/M	diaphyseal	L	no	Partial gastrectomy for gastric heteroplasia, Chronic anemia, Chronic ischemic encephalopathy, Type 2 diabetes mellitus, Hypercholesterolemia, Renal cysts, Smoker	Vertebral D7, D8, D9	−1.5	42.1	28.2	N/A	9.2	1.1
2	100/F	subtrochanteric	L	no	Hypertension, Dementia, Gastroesophageal reflux disease, Psoriasis, Osteoporosis	N/A	N/A	N/A	N/A	243	8	0.8
3	68/F	diaphyseal	R	no	Dyslipidemia, Osteoporosis	no	−3.2	46.5	58.9	54	9.8	0.66
4	91/F	diaphyseal	R	yes	Hypertension, Hypothyroidism, Bilateral Knee Prosthesis	no	−1.4	5.4	93.6	66	8.7	0.58
5	90/F	subtrochanteric	L	no	Heart failure, Hypertension, PSVT, Pulmonary emphysema, Chronic respiratory failure, Stage III CKD Iron deficiency anemia	Humerus Dx	N/A	N/A	156.6	149	8.5	1.82
6	73/F	diaphyseal	R	no	Hypertension, Osteoporosis (treated with Vitamin D, Calcium, and Denosumab)	no	−2.3	23.8	45.8	52	10.3	0.61
7	67/F	subtrochanteric	R	yes	Hypertension, Smoker (20 cigarettes day for 20 years)	Vertebral D11	−3.6	11	74	110	8.3	1.1
8	78/M	subtrochanteric	L	no	Hypertension, Smoker, COPD (Prednisone and Esomeprazole for 5 years)	no	−2.5	5	82	81	8.6	0.9
9	80/F	subtrochanteric	L	no	Gastritis, Depression	Vertebral D12	−3.1	9	66	62	8	0.9
10	74/F	diaphyseal	L	no	Hypertension	no	N/A	6.8	64.3	60	8.9	1.21
11	74/F	diaphyseal	R	yes	No	Vertebral L1	−3.9	9	70	102	8.4	N/A
12	65/M	diaphyseal	L	yes	Chronic ischemic heart disease	no	−3.5	14	70	70	8.9	0.9
13	71/F	diaphyseal	R	yes	No	Distal Radius, Distal Peroneal	−4.7	21	65	66	9.1	0.8
14	66/F	subtrochanteric	L	no	No	no	−3.7	3	85	64	8.5	0.7
15	81/F	diaphyseal	L	no	No	Vertebral D10–D12, Distal Peroneal	−5	8	75	89	8.5	0.9
16	86/F	diaphyseal	L	yes	No	Contralateral hip	−2.6	12	67	79	8.5	1.5
17	82/F	diaphyseal	L	yes	Hypertension, Smoker (30 cigarettes day for 40 years)	no	−3	15	60	105	8.3	1.5
18	80/F	diaphyseal	R	no	Hypertension, Type 1 Diabetes Mellitus	Vertebral L1	−3.6	22	54	91	8.6	0.9
19	80/F	diaphyseal	L	no	Hypertension, Type 2 Diabetes Mellitus	no	−3.5	13	60	N/A	N/A	N/A
20	85/M	diaphyseal	L	no	Chronic kidney disease on dialysis, Hypertensive heart disease, Left endarterectomy, Chronic ischemic encephalopathy post-stroke.	no	N/A	N/A	94.2	86	8.9	5.97
21	77/F	diaphyseal	R	yes	No	no	−4.1	7	76	N/A	N/A	N/A
						**Average**	−3.25	15.20	72.28	90.50	8.74	1.27
						**±SD**	±0.98	±12.14	±25.11	±45.07	±0.57	±1.22

**Table 2:** Descriptive Analysis of AFFs without Bisphosphonate exposure (AFFwB).

AFFwB	BMD	Vitamin D	PTH	ALP	Calcium	Creatinine	Age
N°	17	18	20	18	19	18	21
Missing	4	3	1	3	2	3	0
Mean	−3.25	15.2	72.3	90.5	8.70	1.27	78.5
Median	−3.50	11.5	68.5	80.0	8.60	0.900	80.0
Standard Deviation	0.979	12.1	25.1	45.1	0.478	1.22	8.92
Min	−5.00	3.00	28.2	52.0	8.00	0.580	65
Max	−1.40	46.5	157	243	9.80	5.97	100
Shapiro–Wilk W	0.968	0.801	0.830	0.718	0.936	0.489	0.965
Shapiro–Wilk p	0.773	0.002	0.002	<0.001	0.220	<0.001	0.612

**Table 3:** Principal Component Analysis (PCA) showing the key variables contributing most to total variance: PTH, Vitamin D, BMD, ALP, Calcium, and Creatinine. Missing data were handled using mean imputation. Note: Promax rotation was applied. Abbreviations: PC = Principal Component, ALP = Alkaline phosphatase, PTH = Parathyroid hormone, BMD = Bone Mineral Density.

Component Loadings					
Variable	PC1	PC2	PC3	PC4	Uniqueness
Creatinine			0.946		0.14389
ALP		1.050			0.03096
PTH	−0.441		0.552		0.32348
Vitamin D	1.061				0.06506
Calcium	0.696	−0.415			0.14262
BMD				0.990	0.00382

**Table 4:** Kaiser Meyer Olkin (KMO) Test to determinate sampling adequacy and Barlett’s Test for the significantly. Note: MSA = Measure sampling adequacy.

Test	Result
Overall KMO (MSA)	0.423
MSA by variable	BMD 0.838; Vitamin D 0.394; PTH 0.523; ALP = 0.297; Calcium 0.447; Creatinine 0.514
Bartlett’s Test of Sphericity	χ^2^ = 29.8; Df = 15; *p* = 0.013

**Table 5:** Variance given by principal components. Note: SS loadings = Sum of squared loadings.

Component Statistics			
Component	SS Loadings	% of Variance	Cumulative %
1	1.787	29.8	29.8
2	1.298	21.6	51.4
3	1.213	20.2	71.6
4	0.991	16.5	88.2

## Data Availability

The data are fully available from the corresponding author upon request.

## Supplementary Materials

The following supporting information can be downloaded at https://www.mdpi.com/article/10.3390/jcm15010025/s1, Table S1: Pearson’s (r) and Spearman’s (p) correlation coefficients between clinical and biochemical variables.

## Abbreviations

The following abbreviations are used in this manuscript:

AFF	Atypical Femoral Fracture
AFFB	Atypical Femoral Fracture with Bisphosphonate Exposure
AFFwB	Atypical Femoral Fracture without Bisphosphonate Exposure
ALP	Alkaline Phosphatase
ASBMR	American Society for Bone and Mineral Research
BMD	Bone Mineral Density
COPD	Chronic Obstructive Pulmonary Disease
DEXA	Dual-Energy X-ray Absorptiometry
ICD-9-CM	International Classification of Diseases, Ninth Revision, Clinical Modification
KMO	Kaiser–Meyer–Olkin Test
PCA	Principal Component Analysis
PPI	Proton Pump Inhibitor
PTH	Parathyroid Hormone
RA	Rheumatoid Arthritis
SD	Standard Deviation
