# Supplementary material for: Atypical Femur Fractures Without Bisphosphonate Exposure (AFFwB): A Retrospective Report of 21 Cases
Source: J Clin Med. 2025 Dec 19;15(1):25. doi: 10.3390/jcm15010025 (PMC12786818; doi:10.3390/jcm15010025)
Supplement: Supplementary file 1 [file jcm-15-00025-s001.zip › jcm-4022084-supplementary.pdf]

Table S1. Pearson's (r) and Spearman's (p) correlation coefficients between clinical and biochemical variables.

		T-SCORE	VIT D	PTH	ALP	CALCIUM	CREATININE
**VIT D**	**r di Pearson**	**0.248**	**—**				
	Gdl	19	—				
	value p	0.279	—				
	Rho di Spearman	0.167	—				
	Gdl	19	—				
	value p	0.469	—				
PTH	r di Pearson	-0.130	-0.429	—			
	Gdl	19	19	—			
	value p	0.574	0.052	—			
	Rho di Spearman	-0.205	-0.576 **	—			
	Gdl	19	19	—			
	value p	0.372	0.006	—			
ALP	r di Pearson	-0.080	-0.065	0.291	—		
	Gdl	19	19	19	—		
	value p	0.730	0.780	0.201	—		
	Rho di Spearman	-0.247	0.104	0.208	—		
	Gdl	19	19	19	—		
	value p	0.279	0.655	0.365	—		
CALCIUM	r di Pearson	0.196	0.677 ***	-0.315	-0.537 *	—	
	Gdl	19	19	19	19	—	
	value p	0.396	< .001	0.165	0.012	—	
	Rho di Spearman	0.171	0.402	-0.344	-0.557 **	—	
	Gdl	19	19	19	19	—	
	value p	0.460	0.071	0.127	0.009	—	
CREATININE	r di Pearson	-0.024	-0.044	0.300	0.055	-0.000	—
	Gdl	19	19	19	19	19	—
	value p	0.916	0.849	0.186	0.813	0.998	—
	Rho di Spearman	-0.109	-0.016	0.159	0.525 *	-0.241	—
	gdl	19	19	19	19	19	—
	value p	0.640	0.944	0.491	0.015	0.292	—

Note. * $p < .05$, ** $p < .01$, *** $p < .001$

Pearson's correlation was applied to normally distributed variables, while Spearman's correlation was used for non-normally distributed data (according to Shapiro-Wilk test). Correlation coefficients and p-values are reported ($p<0.005$ considered significant).

https://doi.org/10.3390/jcm15010025
